# SARS-CoV-2 Amino Acid Mutations Detection in Greek Patients Infected in the First Wave of the Pandemic

**DOI:** 10.3390/microorganisms10071430

**Published:** 2022-07-15

**Authors:** Niki Vassilaki, Konstantinos Papadimitriou, Anastasios Ioannidis, Nikos C. Papandreou, Raphaela S. Milona, Vassiliki A. Iconomidou, Stylianos Chatzipanagiotou

**Affiliations:** 1 Laboratory of Molecular Virology, Hellenic Pasteur Institute, 127 Vasilissis Sofias Avenue, 11521 Athens, Greece; nikiv@pasteur.gr (N.V.); raphaelasmilona@gmail.com (R.S.M.); 2 Laboratory of Food Quality Control and Hygiene, Department of Food Science and Human Nutrition, Agricultural University of Athens, Iera Odos 75, 11855 Athens, Greece; kpapadimitriou@aua.gr; 3 Department of Nursing, Faculty of Health Sciences, University of Peloponnese, Sehi Area, 22100 Tripoli, Greece; tasobi@gmail.com; 4 Section of Cell Biology and Biophysics, Department of Biology, School of Science, National and Kapodistrian University of Athens, Panepistimiopolis, 15701 Athens, Greece; npapand@biol.uoa.gr (N.C.P.); veconom@biol.uoa.gr (V.A.I.); 5 Department of Medical Biopathology, Eginition Hospital, Athens Medical School, National and Kapodistrian University of Athens, 72–74 Vasilissis Sofias Avenue, 11528 Athens, Greece

**Keywords:** SARS-CoV-2, mutations, ORF1ab, S protein, M protein, ORF3a, ORF7a, protein structure and stability

## Abstract

Severe acute respiratory syndrome coronavirus 2 (SARS-CoV-2), a novel virus that belongs to the *Coronoviridae* family, emerged in December 2019, causing the COVID-19 pandemic in March 2020. Unlike previous SARS and Middle East respiratory syndrome (MERS) outbreaks, this virus has a higher transmissibility rate, albeit a lower case fatality rate, which results in accumulation of a significant number of mutations and a faster evolution rate. Genomic studies on the mutation rate of the virus, as well as the identification of mutations that prevail and their impact on disease severity, are of great importance for pandemic surveillance and vaccine and drug development. Here, we aim to identify mutations on the SARS-CoV-2 viral genome and their effect on the proteins they are located in, in Greek patients infected in the first wave of the pandemic. To this end, we perform SARS-CoV-2 amplicon-based NGS sequencing on nasopharyngeal swab samples from Greek patients and bioinformatic analysis of the results. Although SARS-CoV-2 is considered genetically stable, we discover a variety of mutations on the viral genome. In detail, 18 mutations are detected in total on 10 SARS-CoV-2 isolates. The mutations are located on ORF1ab, S protein, M protein, ORF3a and ORF7a. Sixteen are also detected in patients from other regions around the world, and two are identified for the first time in the present study. Most of them result in amino acid substitutions. These substitutions are analyzed using computational tools, and the results indicate minor or major impact on the proteins’ structural stability, which could probably affect viral transmissibility and pathogenesis. The correlation of these variations with the viral load levels is examined, and their implication for disease severity and the biology of the virus are discussed.

## 1. Introduction

In December 2019, several cases of hospitalized patients due to pneumonia in Wuhan city caused by a novel virus led to World Health Organization (WHO) declaring this outbreak a pandemic in March 2020 [1,2]. Whole-genome sequencing assisted in characterizing the virus that was named by the International Committee on Taxonomy of Viruses (ICTV) as severe acute respiratory syndrome coronavirus 2 (SARS-CoV-2) [3]. The disease it provokes was named, by the WHO, COVID-19 [4]. The symptoms of the disease are typically mild and self-limiting, including cough, fever, headaches, fatigue, body ache and nausea, and occur 4 to 5 days after exposure [5,6]. Detection of the infected patients is conducted via reverse-transcription polymerase reaction (RT-PCR) on nasopharyngeal swabs [7]. However, especially for people with comorbidities, such as cardiovascular or chronic lung disease and cancer, the symptoms of COVID-19 could easily progress to severe, ultimately leading to respiratory and multiple organ failure [8]. As a result, in order to prevent viral transmission, many countries have implemented strict lockdowns, which, although effective, have a negative socio-economic impact [9,10].

SARS-CoV-2, belonging to the *Betacoronovirus* genus within the *Coronoviridae* family [3], seems to have originated from bats [11] and has been transmitted to humans through pangolins as intermediate hosts [12]. The viral genome is approximately 30 kb [13]. The first 2/3 of the genome is occupied by two overlapping reading frames, ORF1a and ORF1b, which are translated into two polyproteins, pp1a and pp1ab [14,15]. The polyproteins are post-translationally cleaved by viral proteases to sixteen non-structural proteins (Νsps) that are involved in the replication and transcription processes. Translation of ORF1a produces polyprotein 1a ending with Nsp10, followed by the short Nsp11. On the other hand, when a-1 frameshift occurs, the polyprotein 1ab is generated, in which the viral RdRp, Nsp12, is produced after Nsp10 [16]. The remaining part of the genome consists of several small open reading frames that encode four structural proteins—Spike protein (S), Envelope protein (E), Membrane glycoprotein (M), Nucleocapsid protein (N)—and six accessory proteins that are important in pathogenesis mechanisms [17,18]. The virus uses angiotensin-converting enzyme 2 (ACE-2) as a receptor to enter human cells by binding to it through a domain of the viral Spike protein (fragment S1), causing severe acute respiratory syndrome [19].

Phylogenetic studies indicated that lineage A of the virus is the root of the pandemic [20,21]. This occurred along with lineage B, which emerged shortly after. C and D are two main lineages that were characterized after the spread of SARS-CoV-2 but are now considered aliases of lineage B [22]. All other lineages and sub-lineages that have occurred and will occur, as well as their worldwide spread, are documented collectively at https://cov-lineages.org/index.html (accessed on 1 May 2022) [23]. At the moment, the Variants of Concern are considered the Delta variant, which was detected at first in India in late 2020, and the Omicron variant, first identified in South Africa and in Botswana in November 2021. Currently, no SARS-CoV-2 variants are considered Variants of Interest or Variants of High Consequence. The BA.3 lineage and BA.2 lineage with the additional mutation L452X are variants that are being monitored [24].

Although previous SARS and Middle East respiratory syndrome (MERS) outbreaks in 2003 and 2012 had a higher case fatality rate, SARS-CoV-2 is more problematic, since it has a higher transmissibility rate [25,26,27,28]. The more the virus replicates, as it is a very contagious one, the more mutations it is likely to accumulate, having, as a result, a faster evolution rate [29]. Pathogenicity of the virus is clearly affected by the mutations on the virus [30,31,32,33,34,35]. Large-scale studies on SARS-CoV-2 mutations can assist in understanding how they affect viral pathogenesis and drug development against it [36,37]. This is facilitated by the sharing of viral genome sequences in the Global Initiative on Sharing All Influenza Data (GISAID), as well as smaller studies focusing on clinical and epidemiological characteristics associated with specific mutations [31,38,39]. On that note, in this study, we present mutations that were detected within the first months of the viral spread in Greece.

## 2. Materials and Methods

### 2.1. SARS-CoV-2 Isolates

Ten SARS-CoV-2 RNA sequences were isolated from the nasopharyngeal tissue of COVID-19 patients with mild COVID-19 clinical symptoms (non-hospitalized), including cough, sore throat, mild fever below 38 °C and loss of smell, in Athens Metropolitan Region, Greece, between the dates 3 March 2020 and 27 April 2020. The patients were 6 men and 4 women, and their age varied from 5 to 59 years (mean age: 35.8 ± 14.7 years; median age: 39.5 years; IQR: 25.5–42.75).

### 2.2. NGS and Data Analysis

For extraction of viral RNA from Dacron nasopharyngeal swabs, we used the MagCore^®^ Automated Nucleic Acid extractor (RBC Bioscience Corp., New Teipei, Taiwan) with the MagCore^®^ Viral Nucleic Acid Extraction Kit (RBC Bioscience Corp., New Teipei, Taiwan) for 400 µL sample volumes. RNA quantity and quality were determined using NanoDrop2000 (Thermo Fisher Scientific Inc., Waltham, MA, USA). An amount of 5 µL of RNA sample was first used for cDNA synthesis using the QIAseq SARS-CoV-2 Primer Panel kit (Qiagen, Hilden, Germany) followed by PCR using two pools of SARS-CoV-2 primers. The PCR reaction was carried out with an initial holding stage of 98 °C for 2 min and 35 cycles of 98 °C for 20 s, followed by 65 °C for 5 min for annealing and extension. The two PCR products were pooled and purified using 1X PCR Clean DX beads (Aline Biosciences, Woburn, MA, USA). The concentration of purified amplicons was evaluated using the Qubit^®^ dsDNA HS Assay Kit (Life Technologies, Carlsbad, CA, USA). The libraries were prepared using QIAseq FX DNA Library kits (Qiagen, Hilden, Germany) following the manufacturer’s user guide. The 10 ng purified amplicons were used to prepare the libraries. The samples underwent fragmentation, end repair and A-addition followed by adapter ligation with unique indices. Libraries were amplified using adapter specific primers by following the manufacturer’s user guide. Following the library preparation, the final concentration of the libraries was measured using the Qubit^®^ dsDNA HS Assay Kit (Life Technologies, Carlsbad, CA, USA), and the average library size was determined using the Agilent 2100 Bioanalyzer (Agilent Technologies, Santa Clara, CA, USA). The libraries were pooled and diluted (to 0.6 nM) and paired-end sequenced for 500 cycles using the NovaSeq system (Illumina, San Diego, CA, USA). Sequencing was performed at Mr DNA (Mr. DNA lab, Shallowater, TX, USA). 

Next, NGS data were analyzed using the Flomics SARS-CoV-2-analysis pipeline (Flomics Biotech, Barcelona, Spain). The processing pipeline comprised FastQC v0.11.9 (SOURCEFORGE, San Diego, CA, USA) [40] for quality control at the read and sample level, followed by fastp v0.20.1 (GitHub, San Francisco, CA, USA) [41], which performed adapter trimming and discarded low-quality reads. Only the reads with a mean quality threshold above 30 were kept and were passed to Bowtie2 [42], where the remaining reads were aligned versus the Wuhan reference genome (NC_045512.2) [43]. Past this step, reads aligned with Bowtie2 (run with default options, except -D 20 -R 3 -N 0 -L 20 -i S,1,0.50) were input to iVar v1.2.2. (GitHub, San Francisco, CA, USA) [44] to perform variant calling and assign an effect to the genetic variants found in the sample. Only single nucleotide variants and indels with a frequency above 0.5 were reported. Quast v5.0.2 [45] used all the information from the upstream steps to build the genome consensus for the sample, and this consensus was used to assign a viral lineage with Pangolin web tool [46]. The viral lineage was accessed using the GISAID database and PANGO Lineages [47]. In the NGS analysis, depths of less than 10× were identified by read-depth segmentation in an integrated genomics viewer [48]. Data were deposited to the Sequence Read Archive (SRA) under BioProject PRJNA838201. Accession numbers for the viral sequences are presented in Table 1.

### 2.3. SARS-CoV-2 RNA Quantification by Reverse Transcription-Quantitative PCR (RT-qPCR)

Viral RNA was quantified by real-time reverse transcription (RT)-PCR, using the LightMix Modular Sarbecovirus E-gene Kit (Roche, Basel, Switzerland) for detecting the viral envelope protein €-encoding gene, as well as The LightCycler Multiplex RNA Virus Master kit (Roche), according to the manufacturer’s instructions. Myostatin (MSTN) mRNA levels were used as a reference gene, using the LightMix ModularDx Kit MSTN Extraction Control kit (Roche). Positive and negative control samples were analyzed in parallel. The decadic logarithm of −ΔΔCt of viral RNA values of each patient was calculated.

### 2.4. Detection of SARS-CoV-2 Mutations in Sequences Submitted to Online Databases

Mutations detected on the SARS-CoV-2 isolates of the study were searched in the GISAID database to confirm their existence in other viral sequences using CoV-GLUE (https://cov-glue.cvr.gla.ac.uk/, accessed on 25 June 2022). CoV-GLUE is an online web application for the interpretation and analysis of SARS-CoV-2 virus genome sequences, with a focus on amino acid sequence variation. It maintains a database of mutations, insertions and deletions, which have been observed in the GISAID hCoV-19 sequences sampled from the ongoing COVID-19 pandemic. CoV-GLUE was originally developed as part of COVID-19 Genomics UK Consortium using the GLUE framework, a data-centric bioinformatics environment for virus sequence data, at the MRC-University of Glasgow Centre for Virus Research (CVR) and redeveloped in 2021 to scale to the millions of genome sequences available.

### 2.5. Bioinformatics Analysis for Prediction of Protein Structure and Stability

Various bioinformatics tools were utilized to examine the effect of non-synonymous mutations on the structure and stability of ORF1ab (and especially on non-structural proteins Nsp2, Nsp3, Nsp6, Nsp12, Nsp15), ORF3a and M proteins. In the cases where three-dimensional structures of the proteins were available (experimentally determined structures or quality theoretical models), a structure-based analysis was performed utilizing software tools, such as MAESTROweb [49], SDM [50] and Dynamut2 [51]. These tools classify each mutation as stabilizing or destabilizing by providing the predicted ΔΔG, which corresponds to the difference between the predicted folding values of the wild-type and the mutant state of a protein. In particular, MAESTROweb implements a multi-agent machine-learning system and, in addition to the predicted ΔΔG values, it calculates a value of confidence estimation as a prediction quality measure [49]. SDM is a computational approach that is based on environment-specific substitution tables (ESSTs) for the calculation of the stability difference score between the wild-type and mutant protein structures [50]. DynaMut2 is a web server that combines normal mode analysis (NMA) methods to capture protein motion and graph-based signatures for the representation of the wild-type environment in order to investigate the effects of single- and multiple-point mutations on protein stability [43]. The experimentally determined wild-type structures of the proteins were retrieved from Protein Data Bank (PDB) [52]. In cases where there were no experimentally determined structures available, theoretical models built by the D-I-Tasser algorithm (submitted for publication, 2022), which was developed by Yang Zhang’s research group (https://zhanggroup.org/, accessed on 1 May 2022), were used. For the proteins where no structural data were available, the INPS sequence-based method was applied [53,54]. INPS is based on SVM regression and calculates the stability change (ΔΔG) in kcal/mol of single-point mutations in protein sequences [45]. In cases where the mutated proteins resulted from insertions or deletions of amino acids in the wild-type sequence, the PROVEAN web server was utilized [55,56]. PROVEAN calculations are based on BLAST searches and an alignment-based score approach [55] that predicts how a sequence variation affects the function of a protein. It must be noted that the above-mentioned approaches were not applied on the Spike protein of SARS-CoV-2, since there is a wealth of experimental structural data regarding that protein and the mutation of interest (D614G). 

## 3. Results

### 3.1. SARS-CoV-2 Genomes Used in This Study

Ten SARS-CoV-2 genomes were isolated from COVID-19 patients during the first wave of the pandemic in Attica, Greece, and the mutations that were detected on them were analyzed (Table 1). Specifically, all samples were collected between 3 March 2020 and 27 April 2020. Most genomes belonged to lineage A, which is the root of the pandemic, according to the international database https://cov-lineages.org/lineage_list.html (accessed on 1 May 2022), while the rest of them belonged to lineages B.39, which is a USA–UK–Australian lineage, and B.40, which is mainly a UK and Australian lineage with representation in other countries around the world. In these samples, there were 18 distinct mutations detected in total. Two of them were frameshifting mutations caused by nucleotide deletions, five of them were synonymous mutations, while the remaining eleven were missense (amino acid) substitutions.

### 3.2. Mutation Sites in SARS-CoV-2 Genome

**Figure 1:**
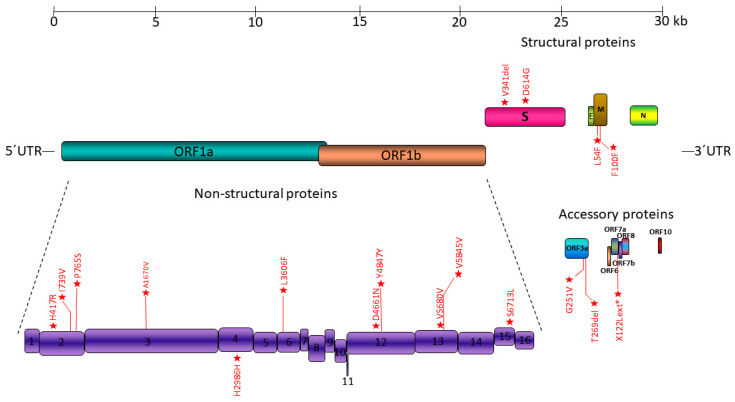
Location of mutations detected on SARS-CoV-2 genomes.

The sequence reads for all samples were mapped to the SARS-CoV-2 genome Refseq (NC_045512), revealing a total of 18 synonymous, non-synonymous amino acid substitutions and frameshifting mutations (Figure 1). These mutations were considered to be real, as they had a frequency of >0.5 on their reads, disfavoring the possibility of being a result of sequencing error.

In detail, regarding the mutations on ORF1ab, which constitutes the larger part of the genome, H417R, I739V and P765S were located on the Nsp2 protein. A1670V was located on the longest SARS-CoV-2 protein, Nsp3. H2986H was found on Nsp4. L3606F was present on Nsp6. D4661N and Y4874Y were located on the Nsp12 protein. On Nsp13, the variations detected were V5680V and V5845V. Lastly, only one mutation, S6713L, was detected on Nsp15.

When it comes to structural proteins, V341del and D614G were mutations located on the Spike protein. L54F and F100F were found on the Membrane glycoprotein. Moreover, regarding the rest of the viral genome, G251V and T269del were detected on ORF3a. Lastly, X122Lext* was located on ORF7a.

### 3.3. Most Common Mutations among the Genotypes Studied

The most common mutations among the samples of the present study were S_D614G and ORF1ab_Y4847Y (Table 1). The spike protein mutation D614G was detected in 5 out of the 10 samples. Y4847Y, which was located at the RNA-dependent RNA polymerase Nsp12 of the virus, was observed in 6 out of 10 samples. In the case of S_D614G, a hydrophilic amino acid was replaced by a hydrophobic one, while ORF1ab_Y4847Y was a synonymous mutation. These two mutations co-existed in only one viral genome (0524-S39). 

Other common substitutions we identified were ORF1ab_L3606F, detected in four samples, and ORF3a_G251V, ORF1ab_I739V and ORF1ab_P765S, observed in three samples each. The two latter mutations co-existed in all three genomes that were present (3396-S31, 9097-S38, 6642-S30). ORF1ab_H417R, ORF1ab_H2986H, ORF1ab_D4661N and ORF1ab_S6713L were detected in two samples each. In ORF1ab_L3606F, ORF3a_G251V and ORF1ab_I739V, hydrophobic amino acids were replaced with amino acids with the same properties. In ORF1ab_P765S, a hydrophobic to hydrophilic substitution was detected. ORF1ab_H2986H was a synonymous mutation. In ORF1ab_H417R, histidine, which has moderate hydropathy, was replaced with a hydrophilic residue. In ORF1ab_D4661N, a hydrophilic amino acid was replaced by a similar one, and lastly, in ORF1ab_S6713L, a hydrophilic amino acid was replaced with a hydrophobic one.

The frequency of the mutations we detected was also searched in a total of 5,228,435 SARS-CoV-2 genomic sequences submitted to the GISAID database, using CoV-GLUE web bioinformatics application. As shown in Table 2, in addition to the ten mutations that we observed in more than one sample, six of the remaining ones, ORF1ab_A1670V, ORF7a_X122Lext, ORF1ab_V5680V, ORF1ab_V5845V, M_F100F and S_V341del, were also present in other viral sequences.

Concerning the mutations ORF3a_T269del (deletion of nucleotide G26199) and M_L54F, this is the first time they have been detected. However, other variations of the specific amino acid residues, T269 in ORF3a and L54 in M protein, have been previously observed in the GISAID submitted sequences (T269del, as a result of whole codon deletion and L54L/L54del, respectively).

### 3.4. Empirical Analysis of Changes in Structure and Stability Parameters

Based on the above data from CoV-Glue, as well as large-scale studies [36,37,57,58,59,60,61,62,63,64,65,66,67,68,69,70,71,72,73,74,75,76,77,78], 10 out of the 11 amino acid substitutions (all except M_L54F) that we identified in the Greek patients’ samples have also been observed in SARS-CoV-2 isolates/genome sequences worldwide, and thus, there is a scientific interest in studying the effects of these mutations on protein stability.

The effect of the 10 non-synonymous (missense) mutations on ORF1ab (Νsp2, Nsp3, Nsp6, Nsp12, Nsp15), ORF3a and M proteins’ structure and stability was studied using various bioinformatics tools described in Section 2.2, and the results are presented below.

In the case of Νsp2, whose functional role is not fully understood [46], two samples presented the ORF1ab_H417R mutation (H237R in Nsp2), while in three samples, we identified the contemporary presence of mutations I739V and ORF1ab_P765S (I559 and P585S in Nsp2, respectively). The experimentally determined structure of Nsp2 is available and deposited in PDB (PDBid: 7MSW). As far as the ORF1ab_H417R (H237R) mutation is concerned, structured-based analysis results by MAESTROweb and SDM indicate that this change does not affect the stability of Nsp2, while Dynamut2 results identify this mutation as destabilizing. At this point, it must be noted that the value of ΔΔG^stability^ calculated by Dynamut2 is −0.16 kcal/mole, indicating that it will not confer significant changes in protein structure. The results of the two contemporary mutations (I739V and P765S) from MAESTROweb, SDM and Dynamut2 conclude that this pair of mutations decreases the stability of Nsp2 but not to a great extent.

Nsp3 is the largest protein of SARS-CoV-2 (1945 aa) and consists of multiple domains, which implies pleiotropic functions [15]. The ORF1ab_A1670V (A852V in Nsp3) mutation was present in one sample and was located in the Nsp3 component that exhibited papain-like protease (PLpro) activity and cleaved polyproteins 1a and 1ab [15,79]. For the analysis of that mutation, we used the structure of papain-like protease Nsp3 that was determined [80] with PDBid 7QCM. The results of MAESTROweb and Dynamut2 agree that this mutation is slightly destabilizing, while SDM predicts the opposite.

For the Νsp6 protein, the information is significantly poor, and there is no experimentally determined structure available to date. It is a transmembrane protein with seven putative trans-membrane helices, as shown by sequence analysis [81], and it participates in protein–protein interactions [80], but the mechanism is not clarified [82]. In four samples, the ORF1ab_L3606F (L37F in Nsp6) mutation was present. Since there is no available structure, the INPS sequence-based method was applied. Our results indicate that L37F causes slight destabilization of Nsp6.

Nsp12 is the catalytic subunit of RNA-dependent RNA polymerase (RdRp) of SARS-CoV-2 [83]. The ORF1ab_D4661N (D269N in Nsp12) mutation was identified in two samples. The structure-based analysis was performed on the determined structure of Nsp12 [84] with PDBid 7C2K. All the methods predicted that the D269N mutation affects the protein structure but not to a great extent.

Two of the studied samples contained the ORF1ab_S6713L mutation in the uridine specific endoribonuclease Nsp15 (S261L), which is used for the cleavage of viral RNA and the evasion of detection by host immune defense systems [85]. The wild-type structure of Nsp15 is determined and deposited in PDB (PDBid: 7N06). Two methods (SDM and Dynamut2) indicate that this is a stabilizing mutation, while MAESTROweb predicts that it is destabilizing.

The rest of the non-synonymous mutations of interests identified in the samples were in the accessory protein ORF3a and the structural proteins M (Membrane protein) and S (Spike protein).

ORF3a is the largest accessory protein of SARS-CoV-2 [86], which is involved in cell death, leading to tissue damage that affects the severity of COVID-19 [87]. The structure determination of the ORF3a protein [88] along with other studies [89,90] reveals that it is a trans-membrane protein with three membrane-spanning helices and a cytoplasmic domain that consists of two beta sheets. These studies also show that the ORF3a assembles into homo-tetramers. In order to perform structure-based analysis of the ORF3a_G251V mutation, which was present in three samples, we downloaded the theoretical model of ORF3a built by the D-I-Tasser algorithm (submitted for publication, 2022), which was developed by Yang Zhang’s research group (https://zhanggroup.org/, accessed on 1 May 2022) because the experimentally determined structures of ORF3a [88] lack the cytoplasmic C-terminal end where the ORF3a_G251V mutation resides. The results of MAESTROweb and Dynamut2 agree that this mutation is highly destabilizing, while SDM predicts the opposite. The INPS sequence-based method verified that this mutation affects the overall structure of the protein.

Membrane (M) protein, which is crucial for both viral infection and host interferon antagonism [81,82], is one of the most conserved proteins of SARS-CoV-2, sharing similar structural and functional characteristics with M proteins from other coronaviruses [91]. It forms homodimers that are essential for the assembly of the virus envelope [92,93,94]. It consists of three domains: an N-terminal ectodomain, three trans-membrane helices and a long C-terminal domain facing the inner side of the virion. One amino acid substitution of interest that was present in one sample, the M_L54F mutation, was also structurally studied, even though it has not been reported elsewhere. Unfortunately, despite the efforts, the experimental structure of the M protein is not yet available. Due to the lack of structure of the M protein, we followed the same approach, which is described for ORF3a, by utilizing a theoretical model built by D-I-Tasser. All the structure-based methods, along with the INPS sequence-based method, agree that this is a mutation that highly affects the structure of the protein. The results of Phobius, a trans-membrane topology prediction method [95], indicate that residue 54 of the M protein resides in its second trans-membrane helix, and, probably, this substitution to a residue with a larger side chain will affect the stability of the protein. The numerical values of the structure-based methods for the studied proteins are presented in Table 3.

In addition to the non-synonymous missense mutations, we also detected the presence of an insertion of amino acid residues in the sequence of ORF7a. Accessory protein ORF7a is a type-I transmembrane protein of 121 aa residues [17] with an anti-IFN-I response function [96]. The X122Lext* variation corresponds to an extension (ext) of the open reading frame by 5 codons (addition of a tail of new amino acids LLNFH), up to the next termination codon, because the termination (X) codon (TGA) of the ORF7a (122nd codon) is converted to a Leucine (L) codon (TTA). The effects of this insertion were calculated by the PROVEAN web server, and its results (PROVEAN score −2.509) indicate that this change is deleterious regarding the function of the protein. However, it must be noted that the results are not quite clear, since the value of the PROVEAN score is almost identical to the cut-off set by the tool (−2.5) to consider a sequence change as deleterious or neutral.

**Figure 2:**
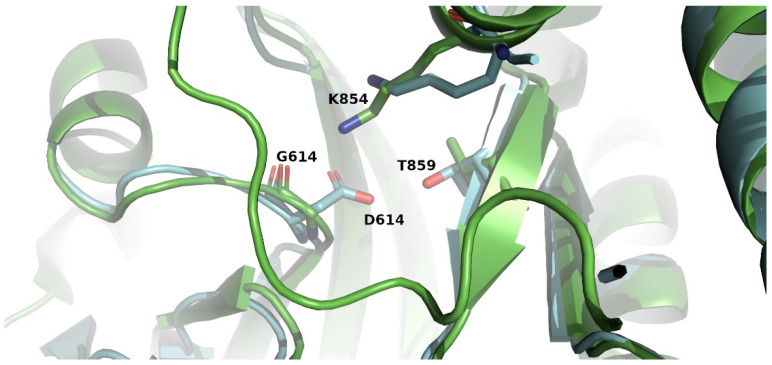
A view of the region where residues D614 and G614 are located in Spike glycoprotein after superposition of the trimeric wild-type colored blue (PDBid 6VSB, [99]) to the D614G trimeric structure colored green (PDBid 7KRQ, [98]). Residues D614, G614, K854 and T859 are presented as stick models. The image was prepared with the molecular graphics software PyMOL (www.pymol.org, accessed on 1 May 2022).

Finally, in five samples, we identified the presence of the S_D614G mutation in the surface glycoprotein Spike (S) of SARS-CoV-2. The S protein is a single spanning membrane protein, which forms a homo-trimer, anchored to the viral membrane by its transmembrane segment [97]. From the available experimental structures of wild-type S protein, it seems that D614 forms a salt bridge with K854 and a hydrogen bond with T859, both in another spike protomer of the trimer, which stabilizes the trimeric structure of the protein, but the D614G substitution results in the loss of these interactions (Figure 2) [64,98] and, consequently, has an impact on its structure.

## 4. Discussion

While the world is still fighting against the SARS-CoV-2 pandemic, genomic studies on the mutation rate of the virus, as well as the identification of mutations that prevail and their impact on disease severity, are of great importance for pandemic surveillance and vaccine and drug development. Previous studies have claimed that the virus is genetically stable [100]. However, we discovered a variety of mutations as early as the first wave of the pandemic, some of which have already been characterized based on their epidemiology and pathogenesis.

In order to evaluate the effect of the discovered mutations on the structure and stability of SARS-CoV-2 proteins (Νsp2, Nsp3, Nsp6, Nsp12, Nsp15, ORF3a and M), we relied on the use of various computational tools that are designed for such analyses and the wealth of previously reported available structural data regarding the virus. Our bioinformatics results indicate that most of the discovered mutations have an effect on the stability of these proteins that is probably connected to a modified protein function and the presence of altered clinical symptoms. The latter has been confirmed in previous studies for a few of the identified mutations, as is mentioned later on. For two of the mutations, S_D614G and ORF3a_G251V, there is previously reported available information on their structural effects on the respective viral proteins. Although thousands of polymorphisms have been identified in genomes of SARS-CoV-2 worldwide, the SNP that corresponds to the D614G mutation presents high frequency ([64]; CoV-GLUE) and has become the dominant strain throughout the world [63]. Residue 614 is located in the C-terminal region of the S1 fragment, outside the receptor-binding domain. Extensive research has revealed an important effect of the S_D614G mutation on the protein structure and function, with a yet unclear mechanism [64,98,101,102,103]. OF3a_G251 residue of ORF3a is located in a region of the cytoplasmic C-terminal end that is conserved, suggesting that its variation may alter ORF3a functional role [90,104,105]. This is consistent with our in silico data predicting a significant effect of ORF3a_G251V substitution on the protein structure and stability. Furthermore, this mutation has been predicted to disrupt the ORF3a–M interactions [105].

Ten out of the eighteen discovered SARS-CoV-2 mutations in our analysis, specifically the amino acid substitutions Orf1ab_H417R, Orf1ab_I739V, Orf1ab_P765S, Orf1ab_L3606F, ORF1ab_D4661N, ORF1ab_S6713L, S_D614G, Orf3a_G251V and the synonymous mutations Orf1ab_H2986H, Orf1ab_Y4847Y, were observed in more than one sample. These specific mutations have also been reported in large-scale meta-analytic studies, where data were extracted from the GISAID (https://www.gisaid.org/), NEXTSTRAIN (https://nextstrain.org/) and National Center for Biotechnology Information (NCBI) (https://www.ncbi.nlm.nih.gov/sars-cov-2/) databases or were tracked during our study in a high number of GISAID SARS-CoV-2 sequences by using the CoV-GLUE web bioinformatics application (https://cov-glue.cvr.gla.ac.uk/, accessed on 25 June 2022) or were published by other groups that used their own large cohorts of patients [36,37,57,58,59,60,61,62,63,64,65,66,67,68,69,70,71,72,73,74,75,76,77,78]. They are either focused on specific geographic regions or on a worldwide scale. Moreover, by using the CoV-GLUE software, six mutations, the amino acid substitutions ORF1ab_A1670V, ORF7a_X122Lext, the synonymous substitutions ORF1ab_V5680V, ORF1ab_V5845V, M_F100F and the frameshifting mutation S_V341del that we identified each in one sample, were also found in the GISAID SARS-CoV-2 sequences (CoV-GLUE Mutations). Finally, the two mutations that were first identified in the present study (ORF3a_T269del, M_L54F) concern residues with previously detected variation (CoV-GLUE Mutations), which could partly explain our findings. These data, including the presence of less common mutations that were identified in the randomly selected SARS-CoV-2 genome sequences of our study and of previous studies with small cohorts of patients [39,106,107,108,109,110,111,112,113,114], further confirm the existence of variation in the genomic sequence, regardless of whether these mutations have been established worldwide producing new viral lineages or not. Viral genome evolution during the first wave of the pandemic has been characterized by the emergence of sets of substitutions that led to the establishment of more than one new viral strain [115,116].

In general, the limited data that have been published about the effect of SARS-CoV-2 amino acid substitutions on the severity of COVID-19 concern mutations that are more common based on the international bibliography. Among these, S_D614G, observed in our study, was not associated with disease severity when examined in a small sample of Egyptian [39] and Chicago [106] patients, in larger cohorts of English patients and in sequences collected online [63,117]. However, one meta-analytic study does report the prevalence of severe outcome in patients that were infected with a SARS-CoV-2 S_D614G mutated variant [61]. In our report, as all patients had mild COVID-19 clinical symptoms, we were not able to deduce any conclusion for the correlation of the specific mutation with disease severity. Moreover, this substitution was previously shown to correlate with increased levels of viral load [63,106,118]. In consistency with these data, a correlation of the presence of S_D614G substitution with the viral load levels in our samples was observed, as the three patients that carried mainly this mutation exhibited among the highest viral RNA amounts in the cohort of patients (Table 1). Concerning the mutation ORF3a_G251V that we identified, two large-scale reports associated it with severe clinical manifestation of COVID-19 [61,119]. Furthermore, ORF1ab_L3606F seems to be associated with asymptomatic cases of COVID-19 in both larger and smaller cohorts of patients [117,120,121] and may correlate with the hypotoxicity of SARS-CoV-2 [62,117,120,121] but was also present in few fatal cases [67].

Efforts in understanding the effect of SARS-CoV-2 mutations on virus biology by utilizing cell cultures and animal model infectious systems have shed light on how some of the mutations we identified could partake in altering mechanisms of virus pathogenicity, infectivity, transmissibility and/or antigenicity. The S_D614G mutation has been associated with higher incorporated levels of the S protein in the viral envelope [101], limited shedding of the S1 domain [101], an alteration of the conformation of the ACE2 binding domain [64,102] and promoted Spike protein lysosomal sorting [103]. Those are some possible explanations for the observed increased virus transmissibility [64,101,103,122,123]. Furthermore, this mutation is located in a B-cell binding epitope and was suspected to negatively impact immunity acquisition after infection or vaccination by causing antigenic drift [39,124]. On the other hand, viral strains containing the S_D614G mutation have been reported as more susceptible to neutralization [123,125,126]. ORF3a_G251V leads to the loss of a B-cell epitope in ORF3a, and thus, it could be related to limited activation of the immune system [127], explaining the clinical manifestations that accompany this mutation. However, the mutation does not inhibit NF-κB activation [127,128]. Lastly, ORF1ab_Y4847Y in nsp12-RdRp has been associated with a higher mutation rate in the membrane glycoprotein (M) and the envelope glycoprotein (E) [58].

Concerning the epidemiological characteristics of the identified mutations, COVID-19 cases bearing the S_D614G mutation were firstly reported in China and Germany, and it appears that it spread from Asia into Europe and the USA [118]. It has become the most common variant since the end of 2019 [59]. The ORF1ab_I739V and ORF1ab_P765S variations, which have been identified in some of the aforementioned large-scale studies [36,57,59,60,65,66,71,72,73,74], have been located in Nigeria [72], England [65,66], Turkey [73], Spain, Asia and USA [66]. They are often found together, proposing that the reason that we identified them in the same viral strains could mean that they entered Greece simultaneously. ORF1ab_H417R, which has been detected in a meta-analysis study, has also been shown to be carried by Brazilian patients [36,107]. ORF1ab_L3606F, which has been described as a homoplastic mutation [75], was observed in China for the first time [59] and has also been reported in studies concerning Italy [59], Brazil [59], Iran [108], Africa [109] and in other studies that do not specify geographic regions [62,67,68,69,120]. Moreover, ORF1ab_Y4847Y, which was not present in China at the early stages of the worldwide viral spread [110], was detected in large-scale studies [59,65,76,77] and placed in Malaysia in the third wave of the pandemic [110], in Thailand in the first wave [111], in Korea [112], in Oceania [77], in Europe [76,77], in Spain, in North America and in Australia [76]. ORF3a_G251V is speculated to have spread at least in January 2020 or earlier in Sweden, Italy, Brazil, Australia and the USA [70], and reached a peak in the months after, followed by its disappearance in April 2021 [113]. In that time window, it was associated with mutations in lineage B [113], explaining, in a way, why we detected it only in viral genomes of this lineage. Generally, it has been identified in worldwide meta-analytic reports [59,60,61,114], in Chinese isolates [105] and in high percentages in England [78]. ORF1ab_H2986H was detected in sewage water in east England [36].

Our study, albeit analyzing a small cohort of patients, contributes to the very few data that have been published about Greece. A total of 41% of transmission in Greece was attributed to imported lineages [129], and viral importation has occurred via multiple routes [130]. This could explain the fact that a lot of the mutations we detected have also been characterized in other regions around the world, as mentioned above. Furthermore, although lineage B.40 has been previously reported to be more frequent in Greece [130], and lineage B.1.17 had the fastest spread [131,132], as compared to lineage A, half of our mutated viral sequences were assigned to lineage A (5/10). The dominant mutation in the first wave of the pandemic was S_G614D [130], in consistence with our observation that it was the most common mutation in our isolates, and has also prevailed in the fourth wave [133]. Regarding the rest of the mutations of our analysis, no references were found in previous data derived from our geographic region. After the first wave, the importation of the virus was limited in our country [129].

We conclude that several mutated variants of SARS-CoV-2 appear to have emerged as early as the first months of the virus spread, which could explain the subsequent establishment of new viral strains and lineages in the population, with different transmission rates, disease severity and escape from neutralizing antibodies. This is a challenge for developing reliable and efficient systems for the detection of the virus and the identification of multiple mutations simultaneously.

## Figures and Tables

**Table 1:** Mutations detected on SARS-CoV-2 genomes.

Sample Name	Accession Number	Age	Gender ^#^	Viral Load Log (−ΔΔCt)	Lineage	Amino Acid (Nucleotide) Mutations
1851-S45	SRR19213734	23	M	3.89	A	S_D614G (A23403G)
4405-S34	SRR19215536	42	M	1.70	A	S_D614G (A23403G), ORF7a_X122Lext * (G27758T)
2384-S29	SRR19215599	45	F	4.13	A	S_D614G (A23403G)
3125-S32	SRR19215604	40	M	2.12	B.39	ORF1ab_H417R or Nsp2_H237R (A1515G), ORF1ab_H2986H or Nsp4_H223H (C9223T), ORF1ab_L3606F or Nsp6_L37F (G11083T), ORF1ab_D4661N or Nsp12_D269N (G14245A), ORF1ab_Y4847Y or Nsp12_Y455Y (C14805T), ORF1ab_V5845V or Nsp13_V521V (A17799G), ORF3a_G251V (G26144T)
3396-S31	SRR19215602	59	M	3.81	B.40	ORF1ab_I739V or Nsp2_I559V (A2480G), ORF1ab_P765S or Nsp2_P585S (C2558T), ORF1ab_Y4847Y or Nsp12_Y455Y (C14805T), ORF1ab_S6713L or Nsp15_S261L (C20402T), ORF3a_G251V (G26144T)
9096-S37	SRR19215566	42	M	−0.69	A	ORF1ab_A1670V or Nsp3_A852V (C3064T), S_F342fs ^#^ (GTT22583G), S_D614G (A23403G), M_L54F (C26682T), M_F100F (C26822T)
9097-S38	SRR19215601	37	F	−0.35	B.40	ORF1ab_I739V or Nsp2_I559V (A2480G), ORF1ab_P765S or Nsp2_P585S (C2558T), ORF1ab_L3606F or Nsp6_L37F (G11083T), ORF1ab_Y4847Y or Nsp12_Y455Y (C14805T), ORF3a_T271del (G26199-ACT)
0524-S39	SRR19215600	39	F	−1.22	A	ORF1ab_L3606F or Nsp6_L37F (G11083T), ORF1ab_Y4847Y or Nsp12_Y455Y (C14805T), ORF1ab_V5680V or Nsp13_V356V (C17304A), S_D614G (A23403G)
2098-S40	SRR19215598	26	M	−1.37	B.39	ORF1ab_H417R or Nsp2_H237R (A1515G), ORF1ab_H2986H or Nsp4_H223H (C9223T), ORF1ab_L3606F or Nsp6_L37F (G11083T), ORF1ab_D4661N or Nsp12_D269N (G14245A), ORF1ab_Y4847Y or Nsp12_Y455Y (C14805T), ORF1ab_V5845V or Nsp13_V521V (A17799G)
6642-S30	SRR19215603	59	M	4.56	B.40	ORF1ab_I739V or Nsp2_I559V (A2480G), ORF1ab_P765S or Nsp2_P585S (C2558T), ORF1ab_Y4847Y or Nsp12_Y455Y (C14805T), ORF1ab_S6713L or Nsp15_S261L (C20402T), ORF3a_G251V (G26144T)

* The substitution of the termination (X) codon (TGA) of the ORF7a with a Leucine (L) codon (TTA) provokes an extension (ext) of the open reading frame by 5 codons (amino acids LLNFH). ^#^ F: Female and M: Male.

**Table 2:** Presence of specific SARS-CoV-2 mutations observed in GISAID sequences, as tracked by performing CoV-Glue web application.

Mutations	ORF1ab Mutations	MutType	C1	C2	C3	Ref-Codon	Mut-Codon	Count	Proportion
Nsp2_H237R	H417R	nonsyn	1514	1515	1516	cAt	cGt	1219	0.000233
Nsp2_I559V	I739V	nonsyn	2480	2481	2482	Att	Gtt	3029	0.000579
Nsp2_P585S	P765S	nonsyn	2558	2559	2560	Cca	Tca	3215	0.000615
Nsp3_A852V	A1670V	nonsyn	5273	5274	5275	gCa	gTa	1428	0.000273
Nsp4_H223H	H2986H	syn	9221	9222	9223	caC	caT	7684	0.00147
Nsp6_L37F	L3606F	nonsyn	11,081	11,082	11,083	ttG	ttT	133,400	0.025514
Nsp12_D269N	D4661N	nonsyn	14,245	14,246	14,247	Gat	Aat	1456	0.000278
Nsp12_Y455Y	Y4847Y	syn	14,803	14,804	14,805	taC	taT	76,696	0.014669
Nsp13_V356V	V5680V	syn	17,302	17,303	17,304	gtC	gtA	136	0.000026
Nsp13_V521V	V5845V	syn	17,797	17,798	17,799	gtA	gtG	3079	0.000589
Nsp15_S261L	S6713L	nonsyn	20,401	20,402	20,403	tCa	tTa	5484	0.001049
S_V341del		del	22,583	22,584	22,585	gTT	g--	13	0.000002
S_D614G		nonsyn	23,402	23,403	23,404	gAt	gGt	5,182,511	0.991216
ORF3a_G251V		nonsyn	26,143	26,144	26,145	gGt	gTt	8069	0.001543
M_F100F		syn	26,820	26,821	26,822	ttC	ttT	8300	0.001587
ORF7a_X122L		nonsyn	27,757	27,758	27,759	tGa	tTa	2104	0.000402

C1, C2 and C3 correspond to one of the three nucleotide positions in each codon. Counts are the number of different viral genomes in the database where each mutation was identified. Proportion refers to the total number of the online submitted sequences to GISAID.

**Table 3:** Results of structure-based methods used for the analysis of non-synonymous mutations identified in the collected samples. Dynamut2: Values of ΔΔG^Stability^ (in kcal/mole) below 0.0 (<0.0) correspond to destabilizing mutations. SDM: Values of ΔΔG_pred_. (in kcal/mole) below 0.0 (<0.0) correspond to destabilizing mutations. MAESTROweb: Values of ΔΔG_pred_. below 0.0 indicate a stabilizing mutation. The values in parentheses correspond to c_pred._, confidence estimation, given as value between 0.0 (not reliable) and 1.0 (highly reliable).

Protein	Mutation	Protein Structure	Dynamut2 (ΔΔG^Stability^)	SDM (ΔΔG_pred._)	MAESTROweb (ΔΔG_pred._)
Nsp2	H417R (H237R) ^#^	7MSW	−0.16	+0.07	−0.020 (0.902)
Nsp2	I739V & P765S (I559V & P585S)	7MSW	−0.28	−2.13 & 0.46	+0.035 (0.902)
Nsp3	A1670V (A852V)	7QCM	−0.58	+1.16	+0.010 (0.923)
Nsp12	D4661N (D269N)	7C2K	−0.21	−0.11	+0.095 (0.862)
Nsp15	S6713L (S261L)	7N06	+0.28	−0.3	+0.006 (0.877)
ORF3a	G251V	D-I-Tasser model	−1.53	+0.31	+0.473 (0.845)
M protein	L54F	D-I-Tasser model	−0.78	−1.31	+2.230 (0.825)

^#^ The numbering of the mutated amino acids outside parenthesis corresponds to the position in the ORF1ab polyprotein before cleavage, while the respective numbering in parentheses corresponds to each Nsp individually.

## Data Availability

All relevant data are within the manuscript.
